# Varying viewpoints of Belgian stakeholders on models of interhospital collaboration

**DOI:** 10.1186/s12913-018-3763-9

**Published:** 2018-12-04

**Authors:** Melissa De Regge, Kaat De Pourcq, Paul Gemmel, Carine Van de Voorde, Koen Van den Heede, Kristof Eeckloo

**Affiliations:** 1 Strategic Policy Cell, Ghent University Hospital, Corneel Heymanslaan 10, B-9000 Ghent, Belgium; 2 Faculty of Economics and Business Administration, Department of Marketing, Innovation and Organisation, Ghent University, Tweekerkenstraat 2, B-9000 Ghent, Belgium; 3 Belgian Healthcare Knowledge Centre, Kruidtuinlaan 55, 1000 Brussels, Belgium; 4 Faculty of Medicine and Health Sciences, Department of Public Health, Ghent University, Corneel Heymanslaan 10, B-9000 Ghent, Belgium

**Keywords:** Collaboration, Networks, Hospitals, Stakeholders, Physicians

## Abstract

**Background:**

Hospitals are increasingly parts of larger care collaborations, rather than individual entities. Organizing and operating these collaborations is challenging; a significant number do not succeed, as it is difficult to align the goals of the partners. However, little research has focused on stakeholders’ views regarding hospital collaboration models or on whether these views are aligned with those of hospital management. This study explores Belgian hospital stakeholders’ views on the factors affecting hospital collaborations and their perspectives on different models for Belgian interhospital collaboration.

**Methods:**

Qualitative focus group study on the viewpoints, barriers, and facilitators associated with hospital collaboration models (health system, network, joint venture).

**Results:**

A total of 55 hospital stakeholders (hospital managers, chairs of medical councils, chair of hospital boards and special interest groups) participated in seven focus group sessions. Collaboration in health care is challenging, as the goals of the different stakeholder groups are partly parallel but also sometimes conflicting. Hospital managers and special interest groups favored health systems as the most integrated form. Hospital board members also opted for this model, but believed a coordinated network to be the most pragmatic and feasible model at the moment. Members of physicians’ organizations preferred the joint venture, as it creates more flexibility for physicians. Successful collaboration requires trust and commitment. Legislation must provide a supporting framework and governance models.

**Conclusions:**

Involvement of all stakeholder groups in the process of decision-making within the collaboration is perceived as a necessity, which confirms the importance of the stakeholders’ theory. The health system is the collaboration structure best suited to enhancing task distribution and improving patient quality. However, the existence of networks and joint ventures is considered necessary in the process of transformation towards more solid hospital collaborations such as health systems.

**Electronic supplementary material:**

The online version of this article (10.1186/s12913-018-3763-9) contains supplementary material, which is available to authorized users.

## Background

Due to financial challenges and the need for high-quality care, collaboration in the hospital sector is changing. In both public and private health sectors, the boundaries between hospitals, and with other sectors, are blurring [1]. The new regulations in the Belgian Minister of Social Affairs and Public Health’s action plan [2] instruct Belgian hospitals to join larger care collaborations, requiring them to join forces to better coordinate and integrate patient care across hospital boundaries and to enhance task distribution. Increased collaboration between Belgian hospitals is deemed necessary, as the Belgian hospital sector is characterized by a high degree of dispersion and fragmentation of both general (e.g., emergency departments [3] and maternity services [4] and more specialized services (e.g., complex cancer surgery [5] and major trauma care [6]). Hospital collaboration is envisaged as one of the instruments that can increase efficiency (e.g., rationing of services and concentration of high-cost services) and quality of care (concentration of services with a clear volume–outcome relationship) [7]. There are many benefits to collaborating with others. In addition to advantages in hospital efficiency, it also allows partners to benefit from each other’s resources and competences and to close communication gaps [7]. Rosco and Pronca (2002) showed that hospitals providing a moderate to high proportion of services at the network or system level were more efficient than hospitals that did not use networks or systems for service provision [8].

Worldwide, hospitals are increasingly parts of larger care collaborations, rather than individual entities. These “partnerships” can take many forms, from informal alliances to formal corporate structures, but all involve the engagement of two or more parties—individuals, groups, or organizations—who agree to work together to achieve a common purpose. Alternative forms of collaboration, such as clinical networks, information sharing, joint treatment or diagnostic centers, new shared assets, and joint facility construction are becoming more popular. The wider range of organizational forms follows the wider range of care delivery models currently being adopted [9]. However, organizing and operating all forms of partnerships and alliances in health care is challenging; a significant number fail [10] and some collaborations perform better than others [11, 12]. The spirit of collaboration and cooperation is constantly under pressure [13]. A major factor inhibiting the success of collaboration, identified by Stegelin and Jones (1991), is the lack of understanding of each involved party’s policies [14]. This is supported by Hine, Fenton, and Custance (2015), who demonstrated that developing a shared vision between all parties is required to make a hospital collaboration work [15]. Collaboration in health care is based on the premise that professionals want to work together to provide better care or to provide the same level of quality of care at lower cost. At the same time, however, they have their own interests and desire to retain a degree of autonomy and independence [16]. As such, as hospitals reshape from individual organizations to larger interhospital collaborations, new challenges to incorporate and involve stakeholders in the collaboration arise. The challenges include understanding the role of key players and adapting the collaboration models to satisfy the needs of the different stakeholders, ensuring that the model fits the need of the collaboration.

However, there is a paucity of research directly relating to the stakeholder view on collaboration and how different collaboration forms and drivers interact in different stakeholder groups. As the influence and power of stakeholders can affect the success of an initiative, their viewpoints on these initiatives are of utmost importance.

Stakeholder relationships have been linked to organizational effectiveness [17] and outcomes [18]. Moreover, stakeholder involvement in strategic decision making on the hospital level has been identified as one of the essential aspects of “good hospital governance” in the future of health care, both internationally [19] and locally [20]. However, different stakeholders may have conflicting interests. Each stakeholder may have different goals: some wish to increase revenue while others aim to decrease costs, illustrating diverging interests, or physicians may be placed in an ambiguous role as hospital managers or clinicians. As an example, Trybou et al. (2011) described how conflicting interests between physicians and hospitals are often cited as a major obstacle to effective collaboration [21].

Studies investigating hospital collaborations in the last two decades have mainly focused on describing them or their reasons for success or failure. Conditions at the outset of collaboration can either facilitate or discourage collaboration among stakeholders [22]. Making the collaborative dynamics clear to participants at the beginning can assist in designing effective and appropriate collaboration forms [23]. It is therefore necessary to choose collaboration forms supported by all stakeholders if hospital collaborations are to work [24–26], and to investigate whether the conflicting interests of different stakeholder groups affect the choice of collaboration form. This study thus aims to empirically explore the viewpoints of hospital stakeholders (hospital managers and chairs of medical councils, hospital boards and special interest groups) on different collaboration models for Belgian hospitals. The range of facilitators and barriers that affect existing hospital collaborations in Belgium has been described by De Pourcq et al. (2018) [27]. In particular, collaborations can be affected by contextual, procedural, and structural factors, which include distance, integrated care level, time needed to make decisions, financial and legal incentives, the level of competition, and the need to align the hospitals’ goals with those of professionals. As such, we also considered the factors affecting hospital collaboration. We examine how diverse these viewpoints are between the different stakeholder groups. Our research questions (RQ) are:

- RQ1: What kind of collaboration model is preferred?
- RQ2: Is the choice of collaboration model influenced by stakeholders’ interests?
- RQ3: What factors affect the hospital collaboration models?

### The role of stakeholders in hospital collaboration

Stakeholder theory provides the grounding for this research by describing the composition of organizations as a collection of various individual groups with different interests. What constitutes a stakeholder is highly contested [28]. Despite the different frames of reference, most scholars agree that the term “stakeholder” refers to “any group or individual who can affect or is affected by the achievement of the organization’s objectives” [29]. Hill and Jones (1992) expanded this definition with “constituents who have a legitimate claim on the firm” [30]. Caroll (1981) also argues that groups or individuals can be stakeholders by virtue of their legitimacy, but broadens the scope of the definition to those who have the ability to affect the organization [31]. Hospital stakeholders have a vested interest in the hospital: these are the organizations, groups, and individuals with a stake in the actions and decisions of the hospital, who can be expected to try to affect those and actions and decisions [32, 33]. For hospitals, stakeholders may be internal, including upper management and physicians, or external, such as employers and government [32].

Emerson et al. (2012) discuss the importance of stakeholders in collaborative governance. Inclusion and diversity give voice to multiple perspectives and different interests, allowing the development of more thoughtful decisions that take a broader view of who will benefit or be harmed by an action [23]. Furthermore, decisions produced through strong engagement processes are fairer, more durable, more robust, and more efficacious [34]. Although decisions should consider the interests of the different stakeholder groups and advance collaboration as much as possible, stakeholders will attempt to affect the decisions and actions of the organization in order to influence the direction of the organization in a way consistent with meeting their needs and priorities (the stakes) [35]. As such, bringing these distinct groups together to reach agreement may not always be possible, so decisions must consider each point of view and optimize decision making to include all voices.

The stakeholder approach extends the traditional management paradigm to include external stakeholders; this is especially significant when stakeholders are active and hospital stakeholder independence is high [36, 37]. As health care organizations operate within increasingly complex networks of relationships, we cannot ignore these interdependencies.

Stakeholder theory suggests that comprehending and then satisfying multiple stakeholder needs can help optimize the effectiveness of the organization and of the network [38–40]. How stakeholders play a role in determining health care organization effectiveness has been described by Fottler et al. (1989) [33] and Blair et al. (1999) [32]; stakeholders affect a range of issues, including financial reimbursement, hospital governance, and patient services. Many different stakeholders have interests in the functioning and outcomes of hospitals [41]. Yet it has been suggested by several studies that groups of healthcare stakeholders can have significantly different perceptions. Differences are particularly evident between managers and clinicians, hospital and nonhospital personnel, health and social service providers, physicians, and other professionals [42–45]. More coordinated and effective action on collaboration can be made possible through a shared understanding of how others think and perform, along with a minimal level of strategic agreement between groups [46–48].

Stakeholder theory is seldom used in nonprofit research [49]. Nonprofit organizations such as hospitals provide a unique context for studying diverse stakeholders and are a relatively unresearched area. Moreover, the diversity of stakeholders in nonprofit organizations is a unique and a complex factor. Studies have however tended to focus on only one stakeholder group in individual organizations [50]. Few studies have focused on the stakeholders’ perspective, so scholars know little about why stakeholders form collaborative alliances or what they think about these hospital collaborations [51]. Moreover, the application of stakeholder theory to hospital collaborations provides an opportunity to explore more complex stakeholder viewpoints (such as special interest groups) than those that are typically represented in stakeholder research (employees).

### Hospital collaboration

Interorganizational collaboration exists in many forms [52]. We can distinguish four different forms of collaboration between hospitals [53]. The least formal and most loosely coupled relationship is *cooperation*, in which “fully autonomous entities share information in order to support each other’s organizational activities” [53]. Accomplishing tasks together suggests a closer affiliation than merely sharing information. When “autonomous groups align activities, sponsor particular events or deliver targeted services in pursuit of compatible goals” parties act in *coordination* [53]. In *collaborations*, parties work collectively through common strategies, each giving up some degree of autonomy as they jointly set and implement goals. A collaboration can be a consortium, a joint venture or a network. The most fully integrated connection, *coadunation*, describes mergers, consolidations and acquisitions, where organizations combine cultures into one unified structure; this is a more radical form of collaboration. Mergers, acquisitions, and health systems are the best-known examples of coadunation [54].

**Fig. 1:**
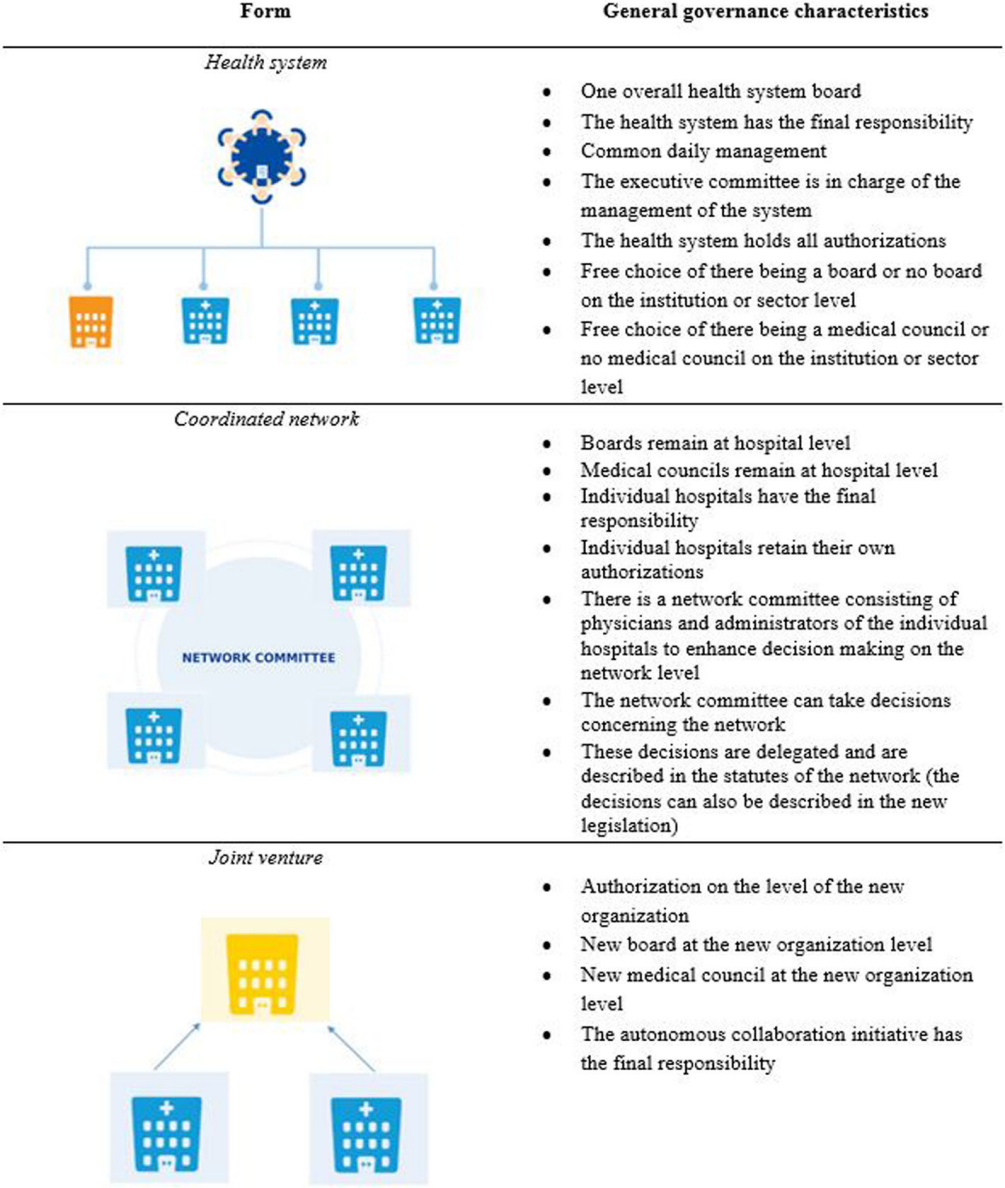
Different collaboration models presented at the focus groups

In this study, we investigate the vision of the different stakeholders regarding different binding forms of collaboration, namely coadunation (i.e. health systems) and collaborations (i.e. networks and joint ventures). These collaboration models vary from very integrated health systems to loosely coupled coordinated networks (see Fig. 1). The models were presented to Belgian hospital stakeholders in order to evaluate their feasibility for each of the collaboration forms.

## Methods

### Case description

The number of collaborations between Belgian hospitals has increased vastly over the last decade. The reasons for collaboration vary and include financial pressure (e.g., the common exploitation of shared services, such as Human Resource Management departments), government regulations, sharing scarce human resources, and providing patient-centered integrated care. Belgian hospitals have recently been required to join larger care collaborations [2]. The current Belgian Hospital Act provides for three types of collaboration between hospitals: “an association (collaboration between two or more hospitals aiming at the joint exploitation of one or more care programs/hospital department or functions), a group (collaboration between hospitals with agreements about task distribution and complementary supply of services, disciplines and equipment in order to meet the needs of the population and to improve the quality of care) and a merger (the most far-reaching form of hospital collaboration since it involves joining two or more hospitals in one hospital with one single administrator)” [55]. According to a current Belgian Health Care Knowledge Centre (KCE) report the collaboration forms in the current Belgian Hospital Act are not sufficient to guide the new developments and new collaboration models are needed [55].

### Study design

In order to evaluate the level of support of the various stakeholders for the proposed collaboration models, a stakeholder consultation was conducted in the form of semi-structured focus groups.

Focus groups are a means of collecting information on a specific subject or area of interest, such as discovery and exploration, or gaining a deeper understanding of how a particular issue is experienced in its context, or to assess the preferences, needs, interests, and attitudes (to health and health care issues) of individuals who have otherwise similar experiences and backgrounds [56]. Focus group participants provide an audience for each other, which encourages a greater variety of communication, and therefore different contents, than other qualitative methods of data collection [57]. This is not the same as the individual semistructured interviews: interaction can help to elucidate understandings and insights, in a ways that questionnaire items and independent questions may be incapable of doing. Focus groups were used here due to our assumption that the moderator–group interaction and the interaction between group members could elucidate some more in-depth responses and reveal perspectives that differ from those drawn out by more carefully designed questions [56, 58].

The relevant aspects of this study are reported following the Consolidated Criteria for Reporting Qualitative Research (COREQ) [59].

### Data collection

The stakeholder consultation was organized through seven focus groups. All Belgian hospitals and special interest groups received an email inviting the different stakeholders. The different stakeholder groups were hospital management (chief executive officers and chief medical officers), chairs of medical councils or their representatives, chairs of hospital boards, and the special interest groups (physicians’ associations from hospitals and primary care, hospital federations, associations of hospital directors, and associations of health care professionals). Stakeholders could then voluntarily participate on one of the proposed dates. There was a response rate of 15% from the hospital managers, 69% from the chairs of medical councils or their representatives, 21% from the chairs of hospital boards and 23% from the special interest groups. The participants were selected on the basis of hospital characteristics (size large or small; private or public; university or not; national, regional, or local; etc.), resulting in a balanced and representative combination of participants from Belgian hospital stakeholder groups.

The focus groups were organized by stakeholder group. Each focus group aimed to include 8–12 participants. It was thought that groups of more than twelve would be too difficult to manage and would hinder meaningful interaction among the participants. In groups smaller than six people, the opportunity for varied inputs is reduced. The focus groups were carried out by:

- Two independent skilled *moderators* (one French-speaking and one Dutch-speaking, the common languages spoken in Belgium) who led and synthesized the discussions to reduce the researchers’ influence.
- An *observer* who helped the moderator to explain the three collaboration models and introduced discussion points.
- Two *reporters* who took notes of the discussion in the group.

A guiding topic list was developed in consensus with the entire research team. This topic list contained the most important questions and prompts for each question (see Additional file 1). Before explaining objectives, the roles of the moderator, observers, and reporters were explained, confidentiality of the discussion was assured, and permission to audio-record the discussion was obtained. The focus group participants were given definitions and explanation of the three collaborative models up front the start of the meeting (see Fig. 1 for the different governance models).

### Analysis

The analysis was carried out by two researchers, both experienced in qualitative research and present at the focus groups, to increase the study’s internal validity and reliability. The focus group meetings were audio-taped and transcribed verbatim. Data were managed and analyzed using NVivo 11.0. We coded the transcripts according to the broad themes of interest of the study (see the topic list) and allowed in vivo codes to arise. The data could then be converted into general viewpoints on hospital collaborations and the pros and cons of each collaboration model according to the stakeholders. In case of disagreements or doubts about interpretation during the analysis, the research team was asked for advice. Finally, the results were verified with the researchers who were present in the focus groups to improve construct validity.

## Results

Seven focus groups were conducted. In some discussions, the number of participants was slightly lower than intended, mostly due to late cancellations or nonappearances. Table 1 provides an overview of the stakeholder type, number of participants, and duration of each focus group.

**Table 1:** Stakeholder type, number of participants, and duration of each focus group

	Stakeholder type	Number of participants	Duration (incl. introduction)
Focus group 1	Hospital managers	12	140 min
Focus group 2	Hospital managers	8	130 min
Focus group 3	Medical council delegates	7	145 min
Focus group 4	Medical council delegates	6	130 min
Focus group 5	Hospital board members	4	130 min
Focus group 6	Hospital board members	8	145 min
Focus group 7	Special interest groups	10	150 min

For RQ1, we investigated the preferred collaboration model. The stakeholders stated that a health system is the preferred model in the long term, but that transition models are also needed.

### Health system

All stakeholders believe that the health system model would increase the quality of care (partly by rationalization and optimization) and improve integrated care by enhancing patient flow through vertical integration. The health system might lower competition between organizations within the system.

*“In a health system rationalizing and optimizing can lead towards better quality.”* Hospital manager [Dutch];

*“To choose for a health system is choosing for a more efficient system.”* Hospital manager [French];

*“A health system aims for quality enhancement through the transmural link, which is very important.”* Chair of hospital board [Dutch];

*“A health system will improve collaboration and transcends the interest of individual organizations.*” Special interest group [French].

However, a health system will acquire a complex governance structure due to the involvement of many different healthcare organizations and large-scale collaborations. Integration into a larger organization might lead to less alignment between the goals of the individual organizations and those of the health system; this might lead to less involvement, causing worse performance and lower motivation at individual hospitals.

*“It’s a complex governance structure which will make it more difficult to involve all participants.”* Chair of medical council [Dutch];

*“Managing a hospital is already very complex without the consideration of other actors, such as elderly care facilities.”* Chair of medical council [French];

*“There is too much diversity between the different organizations today to make a health system work.”* Chair of hospital board [Dutch];

*“It gives the individual hospitals no purpose to perform as an individual, the motivation to perform well will fade.”* Chair of hospital board [French].

Apart from this, smaller organizations inside a health system risk having less decision making rights.

*“Small hospitals will have no say.”* Chair of medical council [French];

*“Will the little ones still play along?”* Chair of hospital board [Dutch];

*“Survival of the fittest.”* Chair of hospital board [French];

*“The ones with exert influence will determine everything.”* Chair of medical council [Dutch].

All stakeholder groups, other than the medical council delegates, pointed out that there might be a risk of patients not being free to choose where they go for health care in the health system collaboration model.

*“You must be able to guarantee the patient's freedom of choice.”* Hospital manager [Dutch];

*“What with the freedom of choice for the patient in this model?”* Hospital manager [French];

*“In the health system the regional freedom of choice of the patient is limited.”* Chair of hospital board [Dutch];

*“The regional freedom of choice of the patient is restricted.”* Special interest group [Dutch].

### Coordinated networks

Coordinated networks were perceived as a very suitable initial collaboration model; it is also a gateway (transition model) towards more integrated collaboration models, such as health systems. This creates extra time for the organization to form collaborations, allowing gradual convergence.

*“Very suitable model for the start of a collaboration.”* Hospital manager [French];

*“Good model to build trust and get to know each other.”* Chair of hospital board [Dutch];

*“It is a gateway to other models, it will create time for the organizations to gradually converge.”* Special interest group [French];

*“A coordinated network in the startup phase is most suitable and achievable. You will evolve from this model towards a health system in the long term.”* Hospital manager [Dutch].

However, the complexity can lead to difficulties managing organizations in the network (decentralized governance, feedback loops, the open system providing too much leeway, weak partnership, individual agreements between individual organizations). Also, a coordinated network might lead to maintenance of the status quo in the collaboration because of lack of task distribution and the rather ‘soft’ feeling of this approach.

*“Feedback to the individual hospitals, I fear, will be unworkable.”* Chair medical council [Dutch];

*“Every decision within the network can be recalled by the individual organization, which is why a network is too soft.”* Hospital manager [Dutch];

*“Rather soft, not sufficiently binding.”* Hospital manager [French];

*“It will be difficult in this model to obtain integration.”* Chair medical council [Dutch].

A positive contrast with the health system is that the governance in the coordinated network works bottom-up which results in greater involvement of all stakeholders and preserves physicians’ independence.

### Joint ventures

Joint ventures were perceived as ideal for technical departments and techniques, such as laboratories and robot surgery, and were also seen as a necessity today. All stakeholders perceived this model as necessary for enhancing the quality of care through specialization. However, they also represent a risk of fragmentation of care. The hospital managers and the hospital board members pointed out that there is a risk of cherry picking in joint ventures: the physicians could select the most profitable services and form joint ventures for these services, perhaps even leave the hospital. This could lead to a loss of revenue for hospitals. They also emphasized that a joint venture is not possible for all kinds of care, as this model is only appropriate for isolated units.

*“A joint venture is an easier solution than a health system or coordinated network. You will be able to supply certain types of care which will provide advantages for the patient, like more focused care, focused procedures.”* Chair medical council [Dutch];

*“An organization focused on a specific pathology or service is needed to enhance quality of care.”* Hospital Manager [French];

*“There is a risk of balkanization, certain specializations will quit the hospital.”* Hospital manager [Dutch];

*“Dangerous for separation of care for the lucrative cases… cherry picking.”* Hospital manager [Dutch];

*“Risk for too much focused care and therefore losing an integrated care, multidisciplinary view.”* Special interest group [Dutch].

For RQ2, we considered whether the choice of collaboration model is influenced by stakeholders’ interests. There are preferences for one of the three models depending on the purpose of the collaboration and the type of stakeholder. The hospital managers and special interest groups favored health systems as the most integrated form that was most likely to enhance collaboration and task distribution. The hospital board members also opted for this model, but believed the coordinated network to be the most pragmatic and feasible model at present. Members of the physician organizations preferred the joint venture, as this creates more flexibility for physicians.

### Health system

The hospital managers affirmed that a health system would improve collaboration and transcend the interest of individual organizations. They emphasized the representation of different health care organizations and the shared resource use. They believed that a health system would decrease excessive competition and duplication of services. Furthermore, the financial responsibility would shift to the health system, providing stability.

*“It is a big advantage that stakeholders of different health care organizations such as home care, elderly care, GPs, are represented.”* Hospital manager [French];

*“As the financial responsibility will be at the highest governance level there will be more stability.”* Hospital manager [French];

*“We will witness a decrease in excessive competition… certainly there will be less duplication of services within the system.”* Hospital manager [Dutch].

However, caution is needed for large-scale collaborations given the risk of monopoly power, a concern that was shared with the special interest groups.

*“Collaboration in one region between all hospitals will give them monopoly in the region, which will lead to lower quality of care.”* Special interest group [French].

Yet a health system is no guarantee of good collaboration between organizations; distance and goal consensus are important to enhance task distribution. According to the medical council delegates, a health system would be especially beneficial if the collaboration is within one region.

*“Equality of the partners is important… Everybody must be able to keep his identity in the health system.”* Hospital Manager [French]*;*

*“A health system must be bound to a region, otherwise it won’t work.”* Chair medical council [Dutch]*.*

A major concern of these delegates was the top-down structure of the health system. They feared that physicians would not be sufficiently involved in the central board that makes general decisions. They doubted whether a distinct centralization of decision making on the collaboration level would be viable, as many medical decisions need to be taken on the hospital level.

*“Too much top-down, too much control from the administrators.”* Chair medical council [Dutch];

*“Participation of physicians in the system might be limited at health system level… the involvement of physicians in decision making in the health system might decrease.”* Chair medical council [Dutch];

*“The organizational culture between the different organizations can be too diverse.”* Chair medical council [French]*;*

*“Centralization leads to a drastic decrease in power of local administrators, there is no equal governance.”* Chair medical council [French].

Also, when other types of organizations (such as nursing homes or primary care organizations) participate, it may prove difficult to align the different stakeholders, and in particular the physicians, of the different organizations - a feeling that was shared by the hospital board members.

*“A difficult collaboration model, as many different organizations, besides hospitals, come together.”* Chair of hospital board [French];

*“Organizations with similar characteristics and features will work well together, but difficulties may be encountered with other type of organizations as ideological origins may differ.”* Chair of hospital board [French];

*“This model will not succeed in the absence of complementarity.”* Chair of hospital board [Dutch].

The members of the hospital boards think that a health system would only be successful if certain conditions were met (e.g., if the ideological stance of all organizations is similar, if all organizations benefit from the health system, and if there is complementarity between the organizations involved).

*“The different organizations are too diverse; there is a need for governance depending on the type of organization.”* Chair of hospital board [Dutch];

*“The organization culture between the different organizations can be too diverse.”* Chair hospital board [French]*.*

The hospital managers proposed some financial risk sharing by all stakeholders (including the physicians) in order to create involvement in management and dedicated decision making.

*“Why not involve the physicians and other stakeholders in the financial responsibility of the organizations or health system? Practically, this can be done by letting them have a say in the care policy and mandating them in the hospital board”* Hospital manager [Dutch].

### Coordinated networks

Hospital managers believe that coordinated networks would be useful if there were little need for guidance, but that the bonds would be weaker than in a health system. Apart from that, this form is mainly focused on the individual advantages of the organization, as collaboration is less strongly encouraged. A major advantage perceived by the hospital management and hospital board members is the freedom to choose partners with the same goals; they do however acknowledge that difficulties can occur when resources need to be allocated between hospitals or when a partner counteracts to the goals and vision of the network.

*“Who will give up their maternity ward if fewer such wards are needed at the network level?”* Hospital manager [Dutch].

The involvement of physicians is necessary to make this form of collaboration work. The medical council delegates indicate that there may be difficulties in aligning the rules that govern physicians. Both the medical council delegates and the hospital board members see an opportunity in the fact that a network allows collaboration even when organizations prefer not to work together on all levels.

*“Possible for the entire hospital or just a service.”* Hospital board member [French].

The special interest groups point out that to make this model work the level of trust between the participants needs to be high, but as it works bottom-up, trust can grow.

*“Level of trust between the participants needs to be high.”* Special interest group [Dutch].

An independent organization to support the network’s administration should be set up.

### Joint ventures

This model is most preferred by the medical boards. They believe that joint ventures are an easier solution than health systems or coordinated networks, and are easy to organize. While the potential risk of cherry picking was not mentioned by the medical boards, this phenomenon was clearly feared by the hospital board members, hospital managers and special interest groups (see also above under results RQ1).

*“Possibility of starting from scratch.”* Chair medical council [Dutch];

*“Possibility of a broad variety of pathologies.”* Chair medical council [Dutch];

*“Task distribution runs smoothly.”* Chair medical council [French];

*“Provides advantages for the patient, like more focused care and focused procedures.”* Chair medical council [Dutch];

*“More independence in the joint venture than in the other models.”* Chair medical council [French];

*“There is a risk of balkanization, certain specializations will quit the hospital.”* Hospital manager [Dutch];

*“Dangerous for separation of care for the lucrative cases… cherry picking.”* Hospital manager [Dutch];

*“Risk for too much focused care and therefore losing an integrated care, multidisciplinary view.”* Special interest group [Dutch].

The other stakeholders pointed out that a joint venture might not be achievable if applied to many different types of care. The special interest groups feared that an excessive amount of focused care, combined with the limited number of these specialized centers, could impede access for patients.

*“The distance for patients might grow with a limited number of very specialized clinics.”* Special interest group [French].

The final research question (RQ3) considered the factors that affect hospital collaboration models, according to the stakeholder groups. All stakeholders supported the idea of forming hospital collaborations. Moreover, they believed that all the collaboration models, if appropriately set up, would be capable of creating the opportunity of enhanced task distribution and collaboration. At the same time they also pointed out that the collaboration form in itself will not resolve all problems related to task distribution and collaboration.

Beyond that, all participants agreed that the three models should be regulated by legislation, as each model reflects a different scope or stage in the collaboration. Although not all collaboration forms were assessed as feasible in the short term, providing the legal opportunity to develop different collaboration forms would allow a natural evolution towards more far-reaching collaboration models, such as full health systems, than is the case today. Therefore, the possibility of combining the three models over time should remain open.

*“You can’t force collaboration, it must grow and needs time.”* Chair of hospital board [Dutch];

*“I think you can evolve from one model towards another, therefore you need different components from different systems.”* Hospital manager [French].

Above that, all stakeholders are requesting parties for a clear regulatory framework to advance collaboration, as for now there are too many impeding factors.

*“We need a regulatory framework in which we can play, we cannot continue now because of obstructive factors.”* Chair hospital board [Dutch];

*“You need clarity in the objectives of the collaboration, we need to have a reason to work together… that should be an obligation from the government.”* Chair Medical Council [French];

*“We need transparency from the legislation, we want to know where we are heading for.”* Hospital Manager [Dutch].

External factors, such as financial reimbursement, also affect the level of collaboration. At the moment, financial incentives are too focused on care in one individual organization, and therefore do not stimulate collaboration; on the contrary, they inhibit it. For now, hospitals are competitors, all seeking a large part of the overall budget. This complicates collaboration and task allocation.

*“The collaboration must be able to determine where the money goes, so at what level the care should be authorized … you have to be able to allocate the cash flow to the highest level of collaboration.”* Chair of hospital board [Dutch];

*“Now hospitals compete for lucrative care, no one will drop these pathologies or procedures. Therefore we should reorganize the financial system to increase quality and enhance collaboration.”* Hospital manager [Dutch];

*“Currently we have problems with how hospitals are financed, this works against networks, nobody wants to hand in care departments if this is linked to less income. That is why the financial framework must change.”* Hospital managers [French];

*“The financial framework inhibits interhospital collaboration.”* Chair of medical council [Dutch].

Communication and trust between the collaboration partners and between the different stakeholders were considered to be the main drivers for collaborations to work.

*“Each successful collaboration starts with trust between the partners.”* Chair of hospital board [Dutch];

*“Trust is the main driver to choose your collaboration partners, therefore we have to be careful in imposing collaborations.”* Hospital manager [Dutch];

*“Most of the collaborations today are being formed from alliances between physicians, physicians that know each other and trust the quality of care that is provided by their colleagues.”* Chair of medical council [Dutch].

The importance of stakeholder involvement and commitment in decision making was also highlighted.

*“One must also listen to the physicians.*” Chair of medical council [Dutch];

*“Involvement of the physicians is needed in all models and on the highest levels.”* Hospital manager [Dutch];

*“From the beginning you have to bring all stakeholders together, because the viewpoints of physicians and administrators will differ. It is important to take into account all the different viewpoints and try to get to an agreement.”* Chair of medical council [French];

*“It is actually the board of directors (hospital board) that makes the decisions, therefore the representation of all stakeholders in the hospital board is important.”* Chair of medical council [French];

*“We must not forget that it is an extra job for the medical board, it comes on top of our clinical work.”* Chair of medical council [Dutch].

There is a trend towards participation of patients and other stakeholders in the governance of health care facilities. As patient participation is increasingly recognized as a key component of the design of health care processes, the stakeholders believe that the voices of the public and patients need to be integrated into decision making in health care facilities and their collaborations.

*“The new collaboration models allow us to rethink the involvement of different stakeholders… In these new models we must certainly listen to the patient. For example representatives of the* *patient support groups should* *be involved in the governance of health care facilities.”* Special interest group [Dutch].

## Discussion

In this paper we looked at the perception of hospital stakeholders on different interhospital collaboration models as stakeholder theory suggests that understanding and meeting multiple stakeholder needs maximizes effectiveness. Emerson et al. (2012) emphasize how engagement and shared motivation stimulate the development of institutional arrangements, leadership, knowledge, and resources, thereby generating and sustaining capacity for joint action (i.e., collaboration) [23]. Thus, engagement and shared motivation are critical in designing the most effective and appropriate form of collaboration. As such, consultation and involvement of the sector (i.e., the various stakeholders) in forming hospital collaborations is essential. Moreover, the alignment of hospital managers and other stakeholders is of major importance for forming successful collaborations [60]. However, this can be problematic due to the diversity of interests among the key stakeholders [39].

All stakeholder groups underscore the importance of shared motivation and values; when the goals of the individual partners in a collaboration are not aligned, or when there is no integrated strategy, the collaboration structure alone will not be able to achieve the aims of the collaboration. Beyond that, the level of trust is indicated as a determinant of collaboration success. This finding supports the results of Provan and Kenis (2008) [61].

According to hospital management, engagement is reflected by the need for stakeholder commitment to the collaboration. This was also found by Johnson et al. (2003) in a study investigating factors related to successful and unsuccessful collaborations [62]. They demonstrated that commitment is a critical factor and the foundation of successful collaborations. Commitment was often missing in unsuccessful collaborations. Their data from stakeholders suggest that if agencies do not have a commitment to the collaboration, the collaboration will probably fail [61]. The hospital managers in this study point out that the sustainability of the organization, in this case the collaboration, is too much the responsibility of the hospital boards. Other stakeholders should therefore share in the risk taking (financially), especially if they want to be involved in the decision-making process. The medical council delegates explicitly asked for involvement at the negotiation phase of the collaboration.

Different preferences for hospital collaborations were identified. As the stakeholder groups favor different collaboration models, the governance and structure of new collaborations will be a challenge. There were preferences for one of the three models depending on the type of stakeholder and purpose of the collaboration. These preferences were in alignment with the interest of the professions. For example, the joint venture model is favored by the physicians, as this organizational form is more focused on one pathology or service, which decreases the complexity in management. Moreover, these types of organizations can function more independently of the hospital. The major concerns about the joint venture for the other stakeholders are that the hospitals might lose their most financially valuable services and that there was a risk of “physician drain” or “cherry picking”. The opinions and visions of the different stakeholders in a collaboration should be aligned. Burns and Muller (2008) mentioned that hospitals need to make efforts to engage physicians by making (among other things) the decision-making processes more participative and responsive [63]. In a health system, the major concern of the physicians is the top-down structure, which implies a loss of decision-making power. As such, physicians should be involved in the policy and should be part of the decision-making processes. This is in line with Trybou et al. (2011) who discuss the importance of physician-hospital alignment, which can not only be realized by “hard” financial means but also needs a more soft sociological perspective, emphasizing the cooperative nature of the relationship [21].

However, the stakeholders also agree on certain topics. Overall, bottom-up decision making that involves stakeholders was identified as very important. Networks and joint ventures have less centralized governance, which leads to the greater involvement of all stakeholder types. On the other hand, a health system is more compelling, although its structure might complicate decision making, which is also a major concern for the physicians.

The stakeholders pointed out that the collaboration model alone will not resolve all problems related to task distribution. External influences also play an important role in the success of the collaboration. Several authors have suggested that it is not the choice of collaboration model in itself that affects collaboration effectiveness [64–67]. Country-specific governance differences in the health care sector are often reflected in variations in the collaboration expectations, structures, and outcomes [68]. That is, government policies make a difference in the ease with which collaborative arrangements are formed and are sustained. According to the participants, the absence of a clear policy framework in Belgium prevents hospitals from taking the next step. A clear framework is needed, but there must be sufficient flexibility for the sector. There are not enough incentives to collaborate, as the current legislation does not financially encourage hospital collaborations (or penalize noncollaboration), and there is no clear vision on how task distribution will be organized. The legislator must therefore provide sufficient flexibility and proper resource planning to make this possible. Flexibility is needed to allow hospitals to choose the best fit between collaboration goals and what is feasible (level of collaboration, level of trust, organizational culture, etc.). Beyond that, the structure of a collaboration should not be fixed, as it can evolve over time [69, 70]. The stakeholders think of the health system as the end point of optimum collaboration. The network can be seen as a transit model to evolve to that optimum and is therefore a necessity in the current health care landscape. It has been indicated in literature that collaboration structures can evolve over time [61]. As such, the views of the stakeholders that different collaboration forms could be combined seem legitimate.

Although this research has provided a perspective on stakeholders’ opinions on hospital collaborations, several limitations need to be addressed. Focus groups are not as efficient as individual interviews in providing maximum coverage of a particular issue. A particular disadvantage is the possibility that the members may not express their honest and personal opinions about the topic at hand. They may be hesitant to express their thoughts, especially when they oppose the views of another participant. The involvement of an expert moderator helped to reduce this problem. As there is no consensus or alignment between different stakeholder groups, future research should investigate how to enhance the interconnectedness of the goals between these stakeholders. A longitudinal study should be conducted to assess the influence of these different stakeholders on the evolution in hospital collaboration models. More quantitative studies on the influence of stakeholders’ characteristics on the type of collaboration could enhance the generalizability of insights of this study.

There is an important role for the patient, as such a more in-depth inclusion of the role and participation of patients in the governance of hospital collaboration might therefore offer further insights on the choice of the collaboration models for hospitals.

## Conclusions

The opinions of the different stakeholder groups run partly parallel, but sometimes they are in conflict. This makes collaborations in health care challenging. The health system was described as the collaboration structure most suited to enhancing task distribution and improving patient quality, but networks and joint ventures should coexist and can serve as transitional steps in an evolution towards a health care system. All structures have both advantages and disadvantages. External factors also affect the collaborations, such as alignment of hospitals’ and professionals’ goals, competition, the financial system and distance. According to the stakeholders, successful collaborations require trust and commitment and governance forms that can change over time.

### Managerial implications

All stakeholder groups perceive decision-making and shared governance as necessary. We recommended that different types of stakeholders, but especially physicians, be involved in all decision-making bodies. Developing a shared vision, and ensuring strategies are aligned with the vision of physicians and other stakeholders, will be critical for successful collaboration.

All stakeholders mention the importance of care delivery focus in integrated health systems, mainly in the long term. In the short term, the coordinated network and/or joint venture can facilitate the collaboration process. This shows the importance of evolution within the collaboration. Managers should be attentive to see opportunities enhancing the collaboration and integrated care.

Also external factors influence the success of collaboration. The current hospital payment system in Belgium complicates interhospital collaborations. The stakeholders also mention that a clear framework is needed, while at the same time there needs to be room for flexibility within the sector. The Belgian government needs to play a major role in facilitating collaboration.

## Additional file

Additional file 1: The topic list contains the most important questions and prompts for each question. (DOCX 14 kb)

## Abbreviations

COREQ: Consolidated Criteria for Reporting Qualitative Research
KCE: Belgian Health Care Knowledge Centre
RQ: Research question
