# Supplementary material for: Varying viewpoints of Belgian stakeholders on models of interhospital collaboration
Source: BMC Health Serv Res. 2018 Dec 4;18:942. doi: 10.1186/s12913-018-3763-9 (PMC6280430; doi:10.1186/s12913-018-3763-9)
Supplement: Supplementary file 1 — The topic list contains the most important questions and prompts for each question. (DOCX 14 kb) [file 12913_2018_3763_MOESM1_ESM.docx]

**Additional file 1**: Topic list

- Support for the 3 models
- Which legal framework is necessary for these models
- For each model
  - Does the model contributes to the objective of the reform (enhance task distribution and increase collaboration)?
  - Will this model be followed?
  - Manageability
  - Collaboration physicians-managers
  - Composition board of directors
  - Other commissions needed
  - Impact on statute of physicians
  - Legal structure and regulations of collaboration
  - Boundary conditions (e.g., authorization, exit rules)
  - Unwanted effects (competition, monopoly, freedom of choice for patients)
  - Collaboration physicians
  - Collaboration outside the models
  - Collaboration with other care facilities (not hospitals)
